# Selective Infection of Antigen-Specific B Lymphocytes by *Salmonella* Mediates Bacterial Survival and Systemic Spreading of Infection

**DOI:** 10.1371/journal.pone.0050667

**Published:** 2012-11-29

**Authors:** Yuri Souwer, Alexander Griekspoor, Jelle de Wit, Chiara Martinoli, Elena Zagato, Hans Janssen, Tineke Jorritsma, Yotam E. Bar-Ephraïm, Maria Rescigno, Jacques Neefjes, S. Marieke van Ham

**Affiliations:** 1 Department of Immunopathology, Sanquin Blood Supply, Division Research and Landsteiner Laboratory, Academic Medical Center, University of Amsterdam, Amsterdam, The Netherlands; 2 Division of Cell Biology, The Netherlands Cancer Institute, Amsterdam, The Netherlands; 3 Department of Experimental Oncology, European Institute of Oncology, Milan, Italy; National Jewish Health and University of Colorado School of Medicine, United States of America

## Abstract

**Background:**

The bacterial pathogen *Salmonella* causes worldwide disease. A major route of intestinal entry involves M cells, providing access to B cell-rich Peyer’s Patches. Primary human B cells phagocytose *Salmonella typhimurium* upon recognition by the specific surface Ig receptor (BCR). As it is unclear how *Salmonella* disseminates systemically, we studied whether *Salmonella* can use B cells as a transport device for spreading.

**Methodology/Principal Findings:**

Human primary B cells or Ramos cell line were incubated with GFP-expressing *Salmonella*. Intracellular survival and escape was studied *in vitro* by live cell imaging, flow cytometry and flow imaging. HEL-specific B cells were transferred into C57BL/6 mice and HEL-expressing *Salmonella* spreading *in vivo* was analyzed investigating mesenteric lymph nodes, spleen and blood. After phagocytosis by B cells, *Salmonella* survives intracellularly in a non-replicative state which is actively maintained by the B cell. *Salmonella* is later excreted followed by reproductive infection of other cell types. *Salmonella*-specific B cells thus act both as a survival niche and a reservoir for reinfection. Adoptive transfer of antigen-specific B cells before oral infection of mice showed that these B cells mediate *in vivo* systemic spreading of *Salmonella* to spleen and blood.

**Conclusions/Significance:**

This is a first example of a pathogenic bacterium that abuses the antigen-specific cells of the adaptive immune system for systemic spreading for dissemination of infection.

## Introduction

*Salmonella enterica* is a Gram-negative, enteric pathogen responsible for diseases that lead to significant morbidity and mortality [1]. After oral uptake, the bacterium crosses the intestinal epithelium via transcytosis of specialized M cells [2] or via luminal capture by sampling dendritic cells [3], [4]. They are eventually internalized by macrophages, dendritic cells and neutrophils in the lamina propia [5], [6]. Cellular entry in non-phagocytic cells is actively induced by the bacterium through an array of effector proteins that orchestrate uptake by manipulating the host’s cellular machinery [7]. *Salmonella* directs host cells during infection to alter the actin cytoskeleton allowing formation of macropinocytic ruffles and entry of the relatively large pathogen into host cells. *Salmonella* introduces bacterial effector proteins in the host cytosol via the *Salmonella* Type III Secretion System (TTSS). *Salmonella* can infect most cell types to form an intracellular vacuole called the *Salmonella*-containing vacuole (SCV). Here, another set of effectors is introduced into the host cytosol for vacuole maintenance and interference with the endosomal system to obtain nutrients and to prevent maturation and fusion with lysosomes [8], [9]. This involves the Akt-AS160-Rab14 cascade and PAK4 [10]. *Salmonella* replicates in an expanding SCV [11], [12] and may thus escape detection by the immune system [13], [14]. Although *Salmonella* replicates in the phagosomes, it remains unclear how the bacteria are released from the infected cell. Obvious mechanisms would involve apoptosis or necrosis of the infected cell, but such is not established.

When *Salmonella* has passed the intestinal epithelium, it spreads via mesenteric lymph nodes to liver, bone marrow and spleen where replication continues [15] and disease ensues. How *Salmonella* reaches these organs is unclear. So far, dendritic cells, macrophages, neutrophils and CD18-expressing phagocytes have been implicated [4], [16]. Neutrophils however exhibit efficient bactericidal mechanisms [6], [17] that render these cells less favorite as vehicles for systemic bacterial dissemination. Similar to HIV (reviewed in [18]), dendritic cells and macrophages may act as pathogen carriers for spreading of the infection, but are unlikely to cause spreading beyond mesenteric lymph nodes. CD11B+ and CD11c+ phagocytes harboring *Salmonella* were reported to be present in blood 5 min after oral inoculation, but it remains to be elucidated how these cells relate to DCs, macrophages or B cells [4], [19].

We recently showed that primary human antigen-specific B cells are able to internalize *Salmonella* after recognition by the B cell receptor (BCR) [20]. As *Salmonella* gains immediate access to the B cell-rich areas of the Peyer’s Patches after intestinal invasion, it may be that B cells mediate spreading of *Salmonella* infection as well. Here we show that *Salmonella* can indeed use antigen-specific B cells as transport vehicle for spreading within the host. *Salmonella* survives intracellularly in a non-replicative state that is actively maintained by the B cell. Ultimately, *Salmonella* is excreted by the B cell followed by reinfection and replication in other cell types. Adoptive transfer of B cells with transgenic BCRs that specifically recognize hen egg lysozyme (HEL)-expressing *Salmonella* showed that *Salmonella*-specific B cells contribute to the *in vivo* systemic dissemination of *Salmonella* in mice after oral administration of the bacteria. The antigen-specific B cells thus act as antigen-specific reservoirs and transport vehicles to release *Salmonella* at distant sites for further infection. These data provide the first example of the use of antigen-specific B cells by a bacterial pathogen for spreading infection in a situation analogous to the involvement of innate cells in spreading of HIV.

## Materials and Methods

### Ethics Statement

All human donors used in this study provided written informed consent in accordance with the protocol of the local institutional review board, the Medical Ethics Committee of Sanquin Blood Supply (Amsterdam, The Netherlands), and the Medical Ethics Committee of Sanquin approved the study. All mice experiments were performed in the European Institute of Oncology, Milan. All experimental procedures using mice were performed according to the Principles of Laboratory Animal Care guidelines (directive 86/609/EEC) and approved by the Italian Ministry of Health.

### Mice

C57BL/6 mice (6–8 weeks old) were purchased from Harlan (Udine, Italy). BCR-HEL VDJ knock-in mice (a kind gift of Dr. J. Cyster, University of California, San Francisco) were bred under specific pathogen-free conditions at Charles River Laboratories. All experiments were performed in accordance with the guidelines established in the Principles of Laboratory Animal Care (directive 86/609/EEC).

### Evaluation of Salmonella Spreading *in vivo*

CD43^-^ naive B cells were purified from spleens of BCR-HEL VDJ knock-in mice with CD43 (Ly-48) Microbeads (Miltenyi Biotec, Bologna, Italy) according to the manufacturer’s instructions. Purity was more than 94%, with more than 75% of the B cells expressing the HEL-BCR. 2*10^5^-10^6^ cells were injected intravenously into C57BL/6 mice (14 mice per group) one day before oral infections. For evaluation of bacterial colonization experiments mice received 6*10^6^ CFUs of HEL surface-expressing *S. typhimurium* SL1344 while transferred B cells were labeled with CFSE (2,5 µM) to monitor their spreading. 0, 24 and 72 hours after infection blood, spleens, mesenteric lymph nodes and liver were collected and processed; a fixed number of cells was lysed with 0.5% sodium-deoxycholate and plated onto TB-agar plates for CFU counting 12 hr later. In spleens and mesenteric lymph nodes the presence of CFSE positive B cells was assessed by FACS at the different time points.

### Antibodies and Bacterial Strains

mAb anti-human IgM (MH15, Sanquin, Amsterdam, The Netherlands) was mixed with rat anti-mouse IgG1 antibody (RM161.1, Sanquin) and mAb anti-*S. typhimurium* LPS (1E6, Biodesign International, Kennebunk, ME) to generate BCR-LPS tetrameric antibody complexes. F(ab)_2_ fragments of MH15 were generated by standard pepsin digestion. Fluorescent secondary antibodies and Texas Red-phalloidin were from Molecular Probes (Leiden, The Netherlands). GFP-*S. typhimurium* SL1344 has been described [21]. The *S. typhimurium* strain 14028 containing the lux operon of *P. luminescens* (luxCDABE) was a kind gift from S. Vesterlund [22] and K. Nealson. Exponentially grown bacteria were washed with PBS, incubated with BCR-LPS tetrameric antibody complexes for 30 min at RT, and washed twice to remove unbound antibodies. Surface HEL-expressing *S. typhimurium* SL1344 was generated by electroporating bacteria with a pVUB4 vector (kindly provided by P. Cornelis, Flanders Institute for Biotechnology, Brussels, Belgium [23]) in which inactive HEL-encoding gene was cloned in frame with the one encoding for OprI protein from *P. aeruginosa* under the control of LacZ promoter. HEL expression was induced by the addition of 1 mmol/L isopropyl-L-thio-B-D-galactopyranoside to exponentially growing bacteria [24].

### Lymphocyte Isolation, Infections and Cell Lines

Human B cells were isolated from peripheral blood of from a buffycoat obtained from healthy donors (Sanquin). This Isolation of human B cells from peripheral blood and culturing of the Ramos B cell line and NIH3T3 fibroblasts expressing human CD40L (3T3-CD40L) have been described [20], purity of peripheral B cells was determined by FACS analysis and was always >99%. B lymphocytes with viable uncoated bacteria and Ramos cells with viable anti-IgM coated bacteria were incubated at 20 bacteria per cell for 40 min at 37°C without antibiotics while tumbling. Next, cells were washed four times and cultured for 1 h in media containing 100 µg/ml gentamicin (Invitrogen) to eliminate non-phagocytosed bacteria. Cells were cultured in RPMI 1640 medium with 5% FCS, p/s, 2 mM L-Glutamine, 50 µM 2-mercaptoethanol, 20 µg/ml human apo-transferrin ((Sigma-Aldrich) depleted for human IgG with prot-G sepharose) and 10 µg/ml gentamicin.

### Live Cell Imaging and EM Analyses

Wide field microscopy was performed at 37°C using 6-well plates (coated with Poly-L Lysine) and a Zeiss Axiovert 200 M microscope equipped with a FluorArc fluorescence lamp, motorized scanning stage, 63× LD Achroplan objective; NA 0.75 and climate chamber. Images were acquired using a Zeiss AxioCam MRm Rev.2 CCD in combination with the manufacturer’s AxioVision software. All experiments presented were repeated several times on different days, and results were consistent and reproducible. Further image processing was performed using the ImageJ software package.

For EM, cells were allowed to take up anti-IgM coated bacteria for 4 hr before fixation in a mixture of paraformaldehyde (4%) and glutaraldehyde (0.5%). After embedding in a mixture of methyl cellulose and uranyl acetate, ultrathin sections were stained and analyzed with a Philips CM electron microscope.

### Intracellular Survival and Growth Assays

Human primary B cells were incubated in parallel experiments with either GFP- or Lux-expressing *Salmonella*. The percentage of living cells and GFP levels were determined using a FACS Calibur (Becton Dickinson). Bioluminescence was measured for 5s in a luminometer (Berthold). Bacterial growth was determined by dividing the relative bioluminescence signal by the relative number of GFP^+^ living B cells, resulting in the amount of light produced per bacteria containing B cell. For induction of apoptosis, cells were treated with 0.1 µM Edelfosine (Biomol) [25].

### Plating Assay

For enumeration of intracellular surviving bacteria, freshly isolated primary B cells were incubated with anti-IgM coated GFP-*Salmonellae* and Ramos cells with uncoated *Salmonellae* as a control, washed and cultured in medium with 10 µg/ml gentamicin as described above. At the indicated time points cells were analyzed by FACS for GFP expression. In parallel, cells were washed with PBS and lysed in 0.1% Triton X-100 (Merck) for 10 min on ice, washed with PBS and a dilution series was plated onto LB agar plates. Plates were incubated overnight at 37°C and colonies were counted.

### Flow Imaging Analysis

Primary B cells were incubated with anti-BCR coated, GFP-expressing *Salmonella* for 1 h in medium without antibiotics, and after washing cultured in the presence of gentamycin. B cell membrane was stained with CD20-PerCP/Cy5.5 (BD) and cells were analysed by ImagestreamX (Amnis) at indicated time points. Analyses were performed using IDEAS software (Amnis). Single cells were gated on *Salmonella* positivity, and only cells and *Salmonella* detected in the focal plane were selected for further analysis. Extracellular and intracellular *Salmonella* were discriminated by deltacentroid-xy analysis, measuring the distance of *Salmonella* to the center of the cell. The numbers of intracellular *Salmonella* were analyzed using the spot-count feature.

### Bacterial Excretion Assay

To visualize bacterial excretion, human primary B cells were incubated with uncoated GFP-*Salmonella* and followed using wide field microscopy in medium containing anti-LPS antibodies coupled to TexasRed. To quantify excretion, cells were stained with DAPI (Sigma-Aldrich) to exclude dead cells and anti-LPS coupled to APC and fixed with 3.7% formaldehyde before analysis using a LSR II (Becton Dickinson). For the increase in LPS levels, the initial level at time point 0 was set to 1. The percentage of excreted bacteria was calculated as the loss of GFP^+^/LPS^-^ B cells compared to time point 0. To discriminate between bacterium and B cell-induced excretion, cells were cultured in medium containing 10 µg/ml tetracycline to arrest intracellular bacteria (bacteriostatic capacity was verified using lux-*Salmonella* in Ramos cells).

### Statistical Analysis

Kaplan-Meier plots and long-rank tests were used to assess survival differences of adoptively transferred mice after virulent *S. typhimurium* infection. Statistic calculations were performed by JMP 5.1 software (SAS, Cary, NC).

## Results

### *Salmonella* Survives in Human Primary Antigen-specific B Cells in a Nonreplicative State

**Figure 1:**
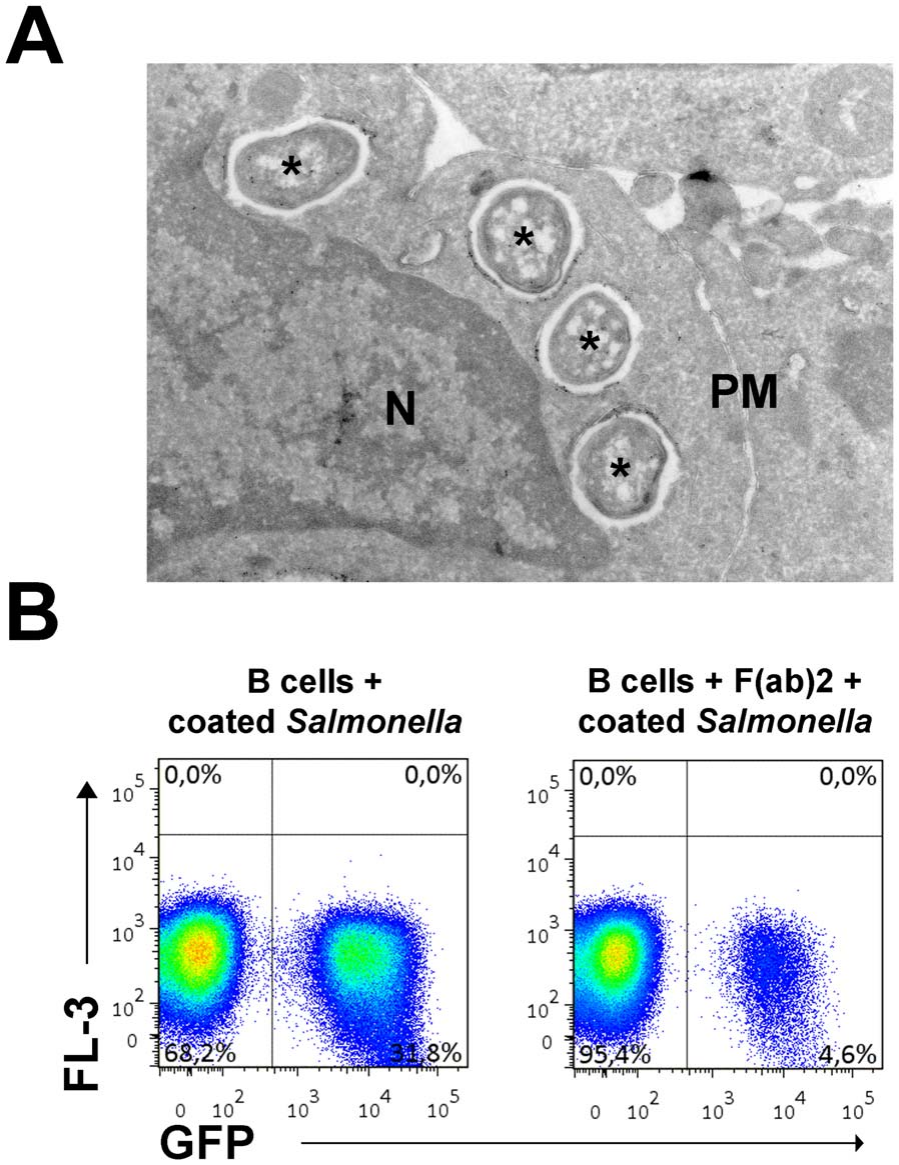
BCR-mediated internalization of *Salmonella* by primary human B cells. (A) Immunocryoelectron micrograph of primary human B cells that had phagocytosed anti-BCR coated *Salmonella*. Gold particles indicate staining for CD63, black asterisks mark bacteria, N marks the nucleus and PM the plasma membrane. (B) Primary B cells were either or not pre-incubated with F(ab)_2_ fragments of the anti-IgM antibody before incubation with live anti-IgM coated GFP-expressing *Salmonella*. After extensive washing, cells were fixed and analyzed by FACS indicating a strong reduction in GFP-*Salmonella* following competition with F(ab)_2_ for BCR interactions. Shown is a representative plot of 5 donors tested.

**Figure 2:**
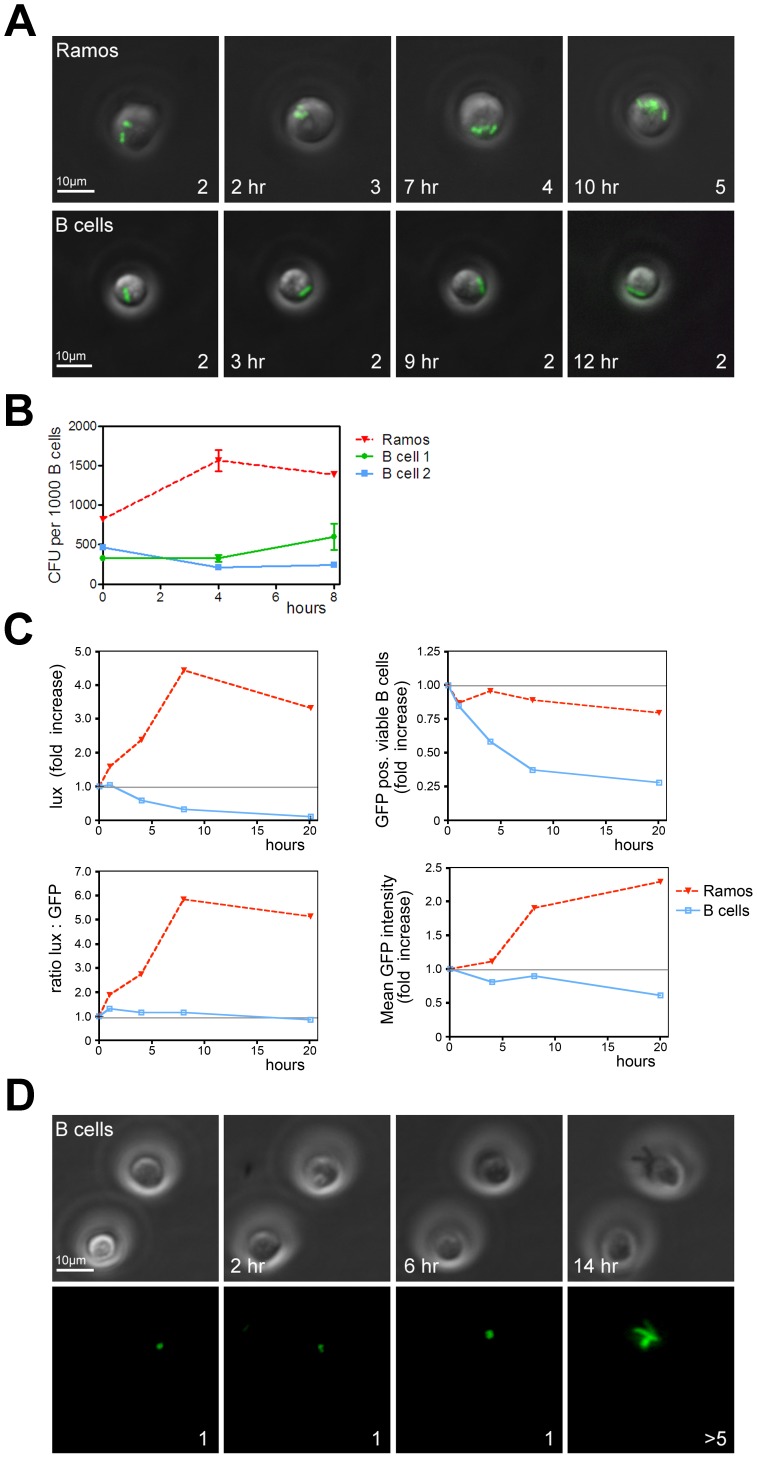
Primary human B cells form a survival niche for intracellular *Salmonella*. (A) Widefield fluorescence microscopy of living Ramos and primary human B cells with phagocytosed anti-IgM coated GFP-*Salmonella*. Depicted are GFP signals projected on the transmission image. Scalebar = 10 µm. Number of bacteria in the visualized cell is given in the lower right corner. Lower left corner: time after *Salmonella* infection. Images are frames from Video S1 and S2. (B) Ramos and primary B cells were incubated with anti-IgM coated *Salmonella*, lysed and plated onto LB-agar plates at various time points after infection. Data are from triplicate experiments performed with Ramos and primary B cells from two individual healthy donors. Error bars indicate SEM. (C) Analysis of Ramos and primary human B cells incubated with living anti-IgM coated lux-expressing (top left panel) or GFP-expressing (top right panel) *Salmonella*. The ratio of lux over GFP shows the amount of light produced per GFP-*Salmonella* positive B cell (bottom left panel), indicating intracellular *Salmonella* viability. The mean fluorescence of the GFP positive population, set arbitrarily at 1 at the beginning of the experiment, shows that the GFP signal increases in Ramos B cells, whereas it decreased in primary human B cells (bottom right panel). A representative example of three independent experiments is shown. (D) B cells were infected with anti-BCR coated GFP-expressing *Salmonella* before exposure to Edelfosine to induce apoptosis. Cells were imaged over a 14 h period. Top panel: transmission image, bottom panel: GFP-signal. Scalebar = 10 µm. Images are frames from Video S3.

We recently demonstrated that primary antigen-specific B cells phagocytose *Salmonella typhimurium* when the bacteria are recognized by the antigen-specific BCR [20]. We now studied the fate of internalized *Salmonella* in more detail. To enhance the number of primary B cells that phagocytose bacteria for some of our experiments, we coated GFP-expressing *Salmonella* with anti-LPS and anti-IgM tetrameric antibody complexes. These complexes bridge the bacterium and the BCR on B cells, leading to BCR crosslinking and uptake of *Salmonella.* Electron microscopy of primary human B cells incubated with *Salmonella* indeed demonstrate the occurrence of phagocytosis as it shows bacteria residing in phagosomes (Figure 1A). Pre-incubation of primary B cells with F(ab)_2_ fragments of the anti-IgM antibody before exposure to anti-IgM coated GFP-*Salmonella* dramatically reduces the amount of GFP-*Salmonella* positive B cells, demonstrating that phagocytosis of *Salmonella* by B cells is mediated by the BCR (Figure 1B). To study the fate of *Salmonella* in B cells over extended periods of time, time-lapse imaging of GFP-tagged *Salmonella* containing B cells was performed using wide-field microscopy to limit phototoxicity. Phagocytosed GFP-*Salmonella* replicated in the Ramos B cell line (Figure 2A, top panel; see also Video S1). Interestingly, no multiplication was detected in human primary B cells (Figure 2A, bottom panel; see also Video S2). This was confirmed by counting of CFU when *Salmonella* was retrieved at various time points from Ramos and primary B cells after uptake of anti-IgM coated *Salmonella* (Figure 2B). These observations show an increase of *Salmonella* numbers over time in Ramos and a steady number of *Salmonella* bacteria in primary B cells. To quantify these observations with more sophisticated methods, we performed parallel experiments to compare GFP-*Salmonella* (detected by flow cytometry) with light producing lux-*Salmonella* (detected by luminometry). Light production by lux-*Salmonella* is dependent on metabolic activity of the bacteria and is thus a marker for bacterial viability [22], while the GFP signal only indicates the cellular presence of the bacteria. GFP-*Salmonella* was used to determine the number of infected B cells at the different time points. Figure 2B shows that the GFP-*Salmonella* phagocytosed by Ramos cells expanded intracellularly. Over a time course of 10 h, the lux activity increased strongly (Figure 2C, top left panel), while the number of Ramos cells positive for GFP-*Salmonella* remained nearly constant (Figure 2C, top right panel). This implies an increase in the amount of light produced per GFP-*Salmonella* positive Ramos cell (Figure 2C, bottom left panel), confirming an increase in numbers of bacteria per Ramos cell over time. In accordance, the GFP signal per Ramos cell increased (Figure 2C, bottom right panel) while lux activity decreased over time when *Salmonella* was phagocytosed by primary human B cells (Figure 2C, top left panel). This was not due to intracellular killing of *Salmonella* as the fraction of GFP-*Salmonella* containing B cells declined equally fast (Figure 2C, top right panel). In fact, the amount of light produced per living GFP-*Salmonella* positive B cell remained constant during the course of the experiment (Figure 2C, bottom left panel), showing that the GFP-positive *Salmonella* remained viable in primary human B cells, albeit under conditions of inhibited replication. Thus, these data confirm the wide-field microscopy data in Figure 2A and show that *Salmonella* does not replicate in primary B cells. Unlike specialized phagocytic immune cells such as macrophages, neutrophils, or B cells from early vertebrates [26], human B cells are apparently inefficient in producing the microbicidal conditions that are required to eliminate *Salmonella*.

We next investigated whether primary B cells actively suppress *Salmonella* growth. We selectively induced apoptosis of human primary B cells (without affecting *Salmonella*; not shown) and measured *Salmonella* replication. Intracellular replication of *Salmonella* was no longer suppressed 2 h after induction of apoptosis in primary B cells with the alkyl-lysophopholipid Edelfosine [25] (Figure 2D; see also Video S3), demonstrating that growth arrest of *Salmonella* requires viable primary B cells. These data suggest that primary human B cells, unlike human B cell lines, actively suppress multiplication of intracellular *Salmonella* within the SCV.

### *Salmonella* is Excreted by Infected B Cells

**Figure 3:**
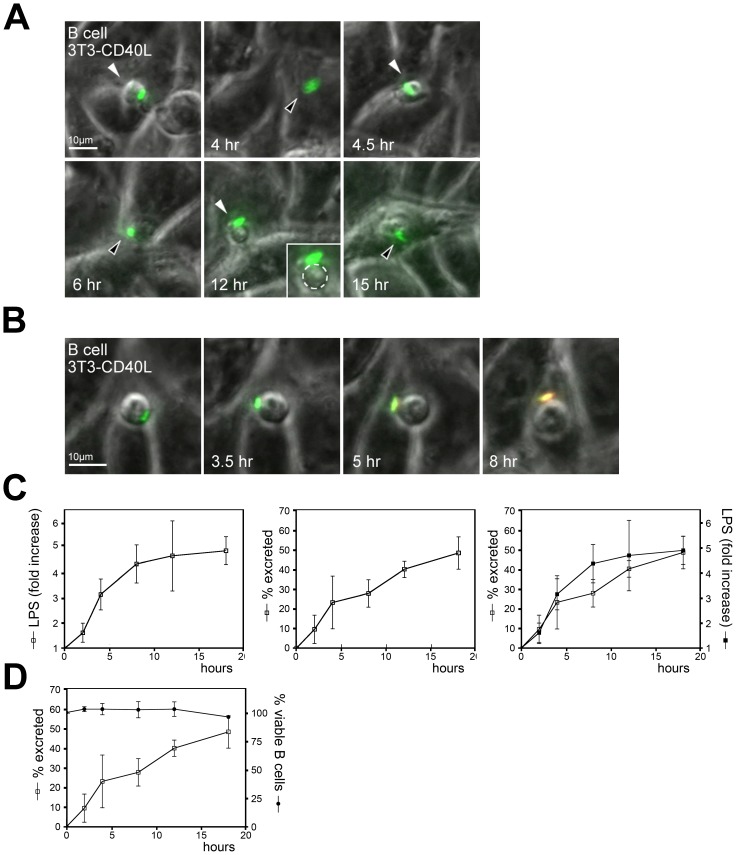
*Salmonella* is actively excreted by B cells. (A) Primary B cells having phagocytosed anti-BCR coated GFP-*Salmonella* on a monolayer of 3T3-CD40L fibroblasts were imaged using widefield fluorescence microscopy. Depicted is the GFP signal projected on the transmission image with images taken every 30 min. Scalebar = 10 µm. Arrows indicate the B cell, white arrow: B cells moves op top of the monolayer, black arrow: B cells moves below the monolayer. Images are frames from Video S4. (B) Primary B cells having phagocytosed anti-BCR coated GFP-*Salmonella* on a monolayer of 3T3-CD40L fibroblasts were imaged using widefield fluorescence microscopy in the presence of TexasRed labeled anti-LPS mAbs. Depicted are GFP and Texas-Red signals projected on the transmission image. Scalebar = 10µm. Images are frames from Video S5. (C) Quantification of *Salmonella* secretion from B cells. Primary B cells were incubated with live uncoated GFP-*Salmonella*. Cells were stained with antibodies against LPS, fixed and analyzed using FACS. Left panel: increase in cell surface exposed LPS from bacteria exposed at the cell surface after initial uptake by B cells. Middle panel: percentage of B cells having excreted *Salmonella* as calculated from the percentage of B cells containing GFP-*Salmonella* followed in time. Right panel: left and middle panels are projected to illustrate that both processes show similar kinetics. Error bars represent SD from three independent experiments. (D) Primary B cells were incubated with live uncoated GFP-expressing *Salmonella* and followed for the time points indicated. The fraction of living B cells is plotted to demonstrate that loss of GFP-*Salmonella* positive B cells is not correlated with cell death.

The observation that the number of primary B cells that had phagocytosed *Salmonella* dropped during prolonged culture of the *Salmonella*-containing B cells (Figure 2B, top right panel) suggested that *Salmonella* might be released from the B cells over time. To visualize the fate of phagocytosed *Salmonella* in B cells, primary human B cells infected with GFP-*Salmonella* were co-cultured on a monolayer of CD40L-expressing 3T3 cells and analyzed by time-lapse wide-field microscopy. Primary B cells that had phagocytosed GFP-*Salmonella* showed extensive invasive behaviour by continuously moving under and over the 3T3-CD40L monolayer (Figure 3A; see also Video S4). At later time points, a fraction of GFP-*Salmonella* appeared to be released from the B cell. To visualize this in more detail, GFP-*Salmonella* infected primary B cells were cultured in the presence of a low concentration of Texas-Red labeled anti-LPS mAb in the medium. GFP-*Salmonella* will attract and concentrate this antibody only upon exposure to the extracellular medium and are excluded from antibody recognition when confined to the B cell interior. Figure 3B shows a B cell with phagocytosed GFP-*Salmonella* that becomes accessible for anti-LPS antibodies in the medium after 5-8 hr of culture (Video S5). *Salmonella* excretion from primary B cells was quantified using FACS by detecting GFP-*Salmonella* and LPS-positive B cells. A strong increase in cell surface exposed LPS on cells that were initially GFP-*Salmonella* positive and LPS negative was observed (Figure 3C, left panel). This suggests that a large fraction of the phagocytosed *Salmonella* were exocytosed as in the example shown in Figure 3B. Accordingly, the population of GFP-*Salmonella* positive/LPS-negative B cells declined over time (Figure 3C, middle panel) with kinetics that were identical to the acquired LPS signal (Figure 3C, right panel), inferring an increased excretion. GFP-*Salmonella* infection of primary B cells did not affect B cell viability or induced apoptosis (Figure 3D). Note that during the first phase of excretion *Salmonella* was released, but remained associated to the B cells, hence the increased staining with the anti-LPS antibodies in the first 10h. The bacterium was later released from the B cell, leveling off further LPS labeling. Loss of the GFP-*Salmonella* signal from infected primary B cells increased over an 18h period in our experiments at which point more than 50% of the bacteria were released from B cells.

**Figure 4:**
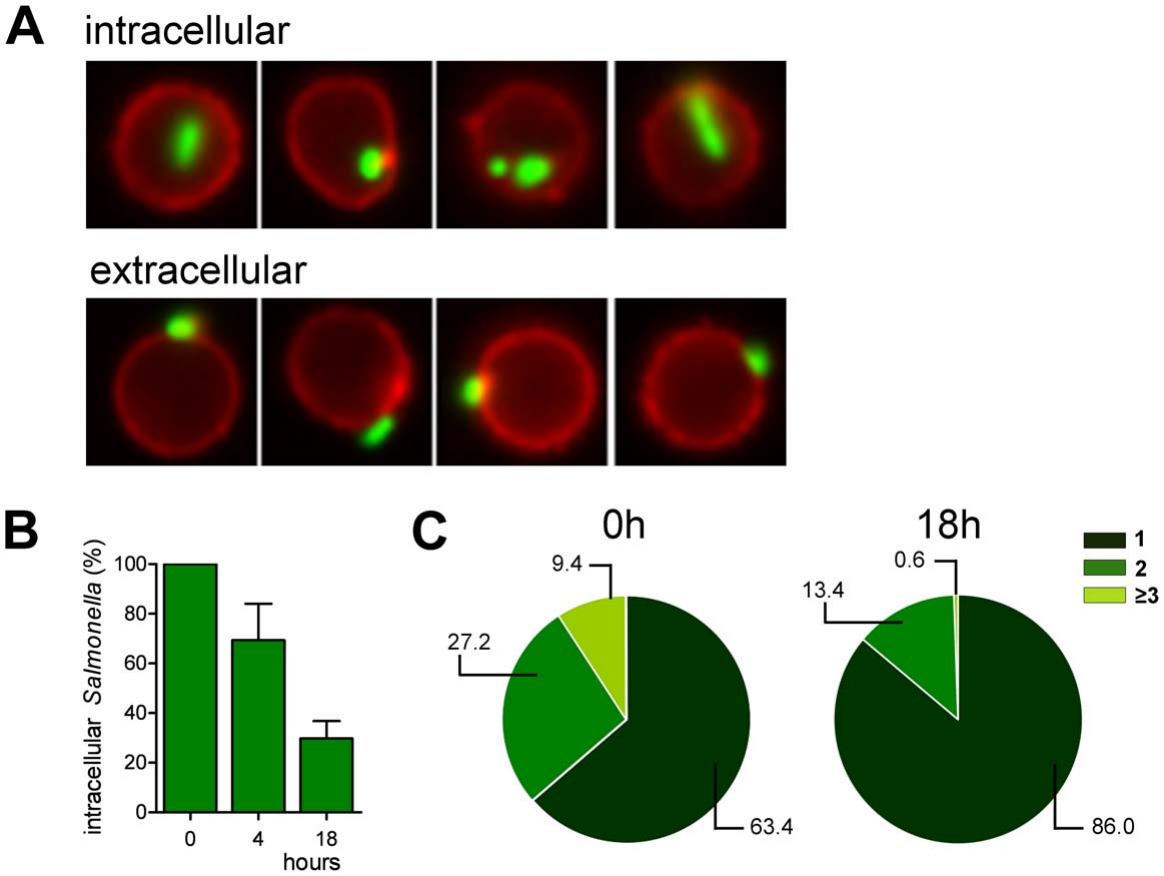
Quantification of the fate of the GFP-expressing *Salmonella* in infected B cells. (A) B cells were infected with anti-BCR coated GFP-expressing *Salmonella* (green).The plasma membrane of the B cells (red) was stained using an anti-CD20 mAb to discriminate between intracellular and extracellular *Salmonella*. (B) The relative amount of intracellular *Salmonella* were measured immediately after infection (0 h), or 4 h and 18 h post-infection. Error bars represent SD from two independent experiments. (C) The number of *Salmonella* per B cell was measured immediately after infection and 18h post-infection. A representative experiment of two individual experiments is shown.

Bacterial excretion was further quantified by Imagestream analyses. This technique directly combines quantitative information obtained by flow cytometry with the visual information of subcellular bacterial localization by microscopy. Specific gating of B cells that had phagocytosed bacteria enabled exact follow-up of B cells with internalized bacteria over time (Figure 4A). Analysis showed a gradual decrease of intracellular *Salmonella* within 18 hrs of infection (Figure 4B). In addition, we tracked the numbers of internalized *Salmonella* per B cell over time, by separating the B cells that had phagocytosed *Salmonella* into different pools based on the GFP-signal per B cell (Figure S1). Immediately after infection, B cells had internalized between 1 and 4 bacteria per cell. Only 1 or 2 bacteria remained per B cell 18 hours later (Figure 4C). In conclusion, our data show that a substantial fraction of phagocytosed *Salmonella* is slowly secreted during the first 24 hrs. This mechanism is not tightly regulated as secretion occurs at an almost linear rate over the first 10 hours of culture post-infection.

### Excretion of *Salmonella* is a B Cell Autonomous Process

**Figure 5:**
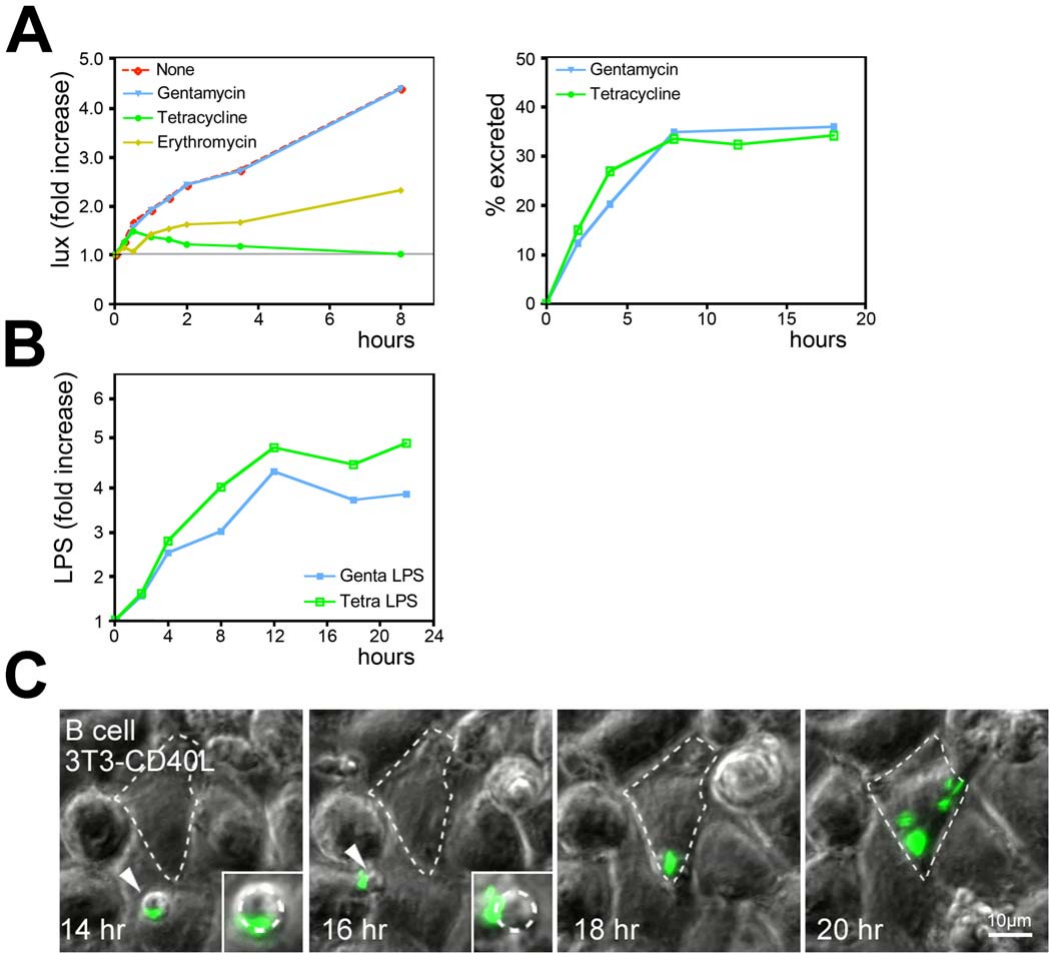
After excretion by B cells, *Salmonella* is capable of infecting secondary host cells. (A) Left panel: the effect of antibiotics on the growth of lux-*Salmonella* in Ramos B cells. Right panel: the same FACS analysis with primary B cells as in 2C was performed in presence of either Gentamicin or Tetracycline to discriminate between host and bacteria mediated excretion. (B) Quantification of *Salmonella* secretion from B cells. Primary B cells were incubated with live uncoated GFP-*Salmonella* in presence of either Gentamicin or Tetracycline to discriminate between host versus bacterial-mediated excretion. Cells were stained with antibodies against LPS, fixed and analyzed using FACS. Increase in cell surface LPS levels is similar in the presence of Gentamicin and Tetracycline, indicating that viable *Salmonella* are not required for excretion. (C) Primary B cells having phagocytosed anti-IgM coated GFP-*Salmonella* on a monolayer of 3T3-CD40L fibroblasts were imaged using widefield fluorescence microscopy for the times indicated. Imaging conditions are similar as in 2A. GFP-*Salmonella* is excreted from a primary B cell (white arrowhead), followed by infection of the 3T3-CD40L monolayer (outline of infected cell marked by a dashed line). Inset shows zoom-in on primary B cell excreting GFP-*Salmonella*. Images are frames from Video S6.

The excretion of *Salmonella* may be controlled by the pathogen or by the B cells. In our experiments we added antibiotic gentamicin at low concentrations to the medium to eliminate extracellular *Salmonella* (and prevent overgrowth of the cells by free *Salmonella*), as gentamicin does not affect intracellular *Salmonella* replication (see Figure 2A). Unlike gentamicin, the antibiotics tetracycline and erythromycin are able to enter phagosomes and eliminate intracellular *Salmonella* [27]. We validated this by testing growth of lux-*Salmonella* in Ramos cells. Tetracyclin and erythromycin inhibited intracellular *Salmonella* growth in Ramos cells (Figure 5A, left panel). In addition, no viable *Salmonella* bacteria were recovered in plating assays of infected B cells, while bacterial colonies were obtained from *Salmonella*-containing B cells exposed to gentamicin (data not shown). *Salmonella* secretion by primary B cells was measured in the presence of either gentamicin or tetracycline to test whether viable bacteria were required for B cell secretion. Tetracycline did not affect excretion of GFP-*Salmonella* from primary B cells. In fact, this occurred equally efficient as B cell excretion in the presence of gentamicin (Figure 5A, right panel), indicating that viability of *Salmonella* is not required for excretion. This was further confirmed by the observation that cell surface LPS levels increased at a similar rate when infected B cells were cultured in the presence of tetracycline as in the presence of gentamicin (Figure 5B). While *Salmonella* actively participates in uptake after capture by the BCR [20], excretion does not require viable *Salmonella*.

### Excretion of *Salmonella* from B Cells Allows Reproductive Reinfection of Other Cell Types

As *Salmonella* survives within antigen-specific B cells, *Salmonella* could also infect other cell types when released from B cells at distant sites. We co-cultured primary human B cells containing phagocytosed GFP-*Salmonella* on a monolayer of 3T3-CD40L and followed the behavior of *Salmonella* using time-lapse wide-field microscopy. Figure 5C shows an example of a phagocytosed GFP-*Salmonella* entering the field of imaging. The GFP-*Salmonella* is released from the B cells and infects the underlying fibroblast monolayer, followed by rapid expansion inside these fibroblasts (see Video S6). The released bacteria not only infected but also resumed replication in the fibroblast monolayer, demonstrating that its passage through primary B cells had not disabled bacterial replication in an irreversible manner. Collectively these data suggest that *Salmonella* can use primary *Salmonella*-specific B cells as a survival reservoir and transport vehicle allowing escape from immune attack and transfer to distant locations.

### *Salmonella*-specific B Cells Mediate Spreading in Acute *in vivo* Infection

**Figure 6:**
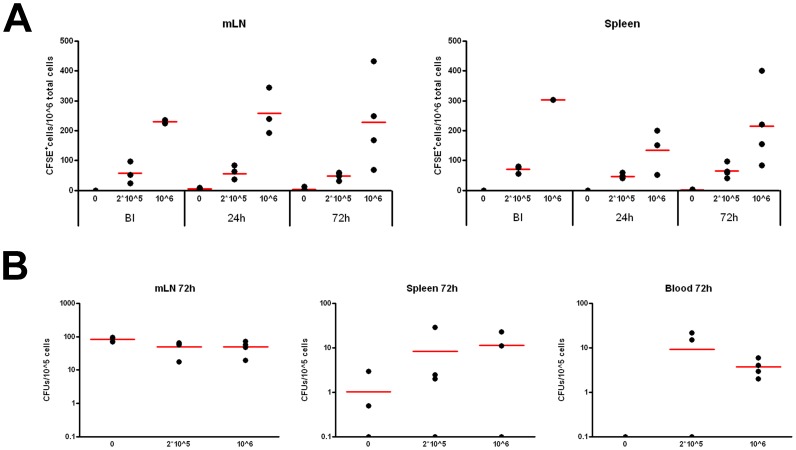
*Salmonella*-specific B cells form a survival niche supporting in vivo *Salmonella* spreading to systemic sites. C57BL/6 mice were adoptively transferred with 0, 2*10^5^ or 10^6^ HEL-specific CD43- naïve B cells labeled with CFSE, as indicated. Mice were orally infected with surface HEL-expressing *Salmonella* one day after B cell transfer. (A) Distribution of CFSE-labeled HEL-specific B cells in the mesenteric lymph nodes (mLN) and spleen before infection (BI), and 24 or 72 hours post-infection, as indicated. One representative example from 3 experiments with 4 mice for each experimental setting is shown. (B) Recovery of viable bacteria 72 hours post-infection from mesenteric lymph nodes (mLN) spleen (SP) and blood (BL) in infected mice transferred with 0, 2*10^5^ or 10^6^ HEL-specific B cells. Depicted are colony-forming units (CFU)/10^5^ eukaryotic cells. One representative example from 3 experiments with 4 mice for each experimental setting is shown.

Our observations imply that the availability of B cells with pathogen-specific BCRs may support spreading of infection when used as carriers for *Salmonella*. *In vivo*, *Salmonella* first encounters B cells after crossing the intestinal epithelium via the M cells. The M cells are directly located over the gut-associated lymphoid tissue (GALT) sites where many B cells reside in Peyer’s patches. Among preferred distant sites of persistent infection for *Salmonella* are the spleen and mesenteric lymph nodes. Indeed, *Salmonella* has been isolated from splenic macrophages and splenic B cells of orogastrically infected mice [28]. How *Salmonella* spreads from the GALT to peripheral compartments is unclear. Transport of viable *Salmonella* by neutrophils [16] is probably inefficient given their efficient bactericidal capacity [5]. Also macrophages and DCs have been implicated, but their spreading beyond the mesenteric lymph nodes may be limited. Antigen-specific B cells as transport vehicles have not been considered. To test this option *in vivo*, different numbers of CD43^-^ naive murine B cells (94% pure preparation) carrying a BCR specific for the HEL antigen were adoptively transferred into WT C57BL/6 mice. One day after transfer, mice were orally infected with surface HEL-expressing *Salmonella*. To directly establish if HEL-specific B cells circulate and mediate systemic dissemination of HEL-expressing *Salmonella*, we investigated both B cell and bacterial recovery from mesenteric lymph nodes, spleen and blood in the different experimental settings, 24 and 72 h after oral infection. Various organs or blood were collected and cells and *Salmonella* isolated. Transferred CFSE-labeled B cells were detected in the mesenteric lymph nodes and spleen and there were no signs of evident expansion (Figure 6A). *Salmonella* infection did not affect HEL^+^-B cell recovery from the organs. Lysis of organs recovered from infected mice followed by plating of the bacteria revealed the number of Colony Forming Units (CFU) in each compartment. Oral infection of mice with HEL-expressing *Salmonella* without adoptive B cell transfer resulted after 72 h after infection in prominent infiltration of the mesenteric lymph nodes and limited spreading to the spleen (Figure 6B). No bacteria were detected in circulating blood cells at the time of sampling, which points to low numbers of *Salmonella* infected cells in blood or a relative short transit time of the cells. Transfer of HEL-specific B cells mildly reduced bacterial dissemination to the mesenteric lymph nodes, but substantially enhanced spreading of *Salmonella* to the spleen (Figure 6B). In addition, *Salmonella* was now retrieved systemically from circulating cells in the blood compartment (Figure 6B).

These data suggest that -in this oral-infection model- antigen-specific B cells do not only promote local spreading of *Salmonella* to the mesenteric lymph nodes, but strongly support systemic dissemination of *Salmonella* into the circulating blood pool and spleen. Pathogen-specific B cells thus form a novel example of immune cells that can be captured by bacteria for spreading and infection of distant sites.

## Discussion

Recent data indicated that B cells from early vertebrates act as efficient phagocytes unlike mammalian B cells that did not show phagocytic behavior [26]. We demonstrated that human B cells have not lost this phagocytic capacity but require the BCR for phagocytosis of particles or pathogens [20]. Phagocytosed *Salmonella* infects and grows in many cell types, but can only be efficiently destroyed in specialized cells like macrophages and neutrophils by the NADPH-oxidase system [5]. A recent report showed also internalization of *Salmonella* in murine B cells, and demonstrated intracellular survival of *Salmonella* [29]. We previously noticed survival of *Salmonella* in B cells after BCR-mediated internalization and now studied the fate of these phagocytosed *Salmonellae* and the consequences for dissemination of infection *in vivo*.

We demonstrated that *Salmonella* can be excreted from primary B cells in time. The factors controlling *Salmonella* excretion are unclear. Our data suggest that the B cell rather than the bacterium controls this process, as both living and dead *Salmonella* can be excreted. Since many bacterial pathogens manipulate the host cell biology for survival, uptake by pathogen-specific B cells followed by release of the pathogen at distant sites may be a more general mechanism for pathogen spreading. Identification and manipulation of signaling pathways to prevent excretion would be a possible means to limit systemic spreading of *Salmonella* and potentially of other pathogens by B cells.

Does BCR-mediated immune escape and spreading by B cells play an important role during *Salmonella* infection? The bacteria may potentially encounter specific B cells very early during infection as they cross the intestinal epithelium and enter the GALT sites. Among preferred sites of persistent infection are localized mesenteric lymph nodes and the more distant spleen. *Salmonella* was thought to reach these locations after transport by neutrophils [16], a notion difficult to match with the efficient bactericidal capacity of neutrophils [5]. Our experiments indicate that *Salmonella*-specific B cells act as transport carriers for the *in vivo* spreading of *Salmonella* to the distant sites. In the event of excretion in B cell rich areas like the spleen and GALT, the bacterium might even go through multiple rounds of uptake (by other resident *Salmonella*-specific B cells), dissemination, and excretion. *Salmonella* thus abuses the specificity of the adaptive immune system to hide from the early innate immune defenses while hitch-hiking inside the B cell ensures systemic spreading of the infection. Dissemination of *Salmonella* to mesenteric lymph nodes does not require B cells, consistent with the concept that *Salmonella*-infected DCs can reach the mesenteric lymph nodes and may thus mediate this localized transport [3], [4], [30].

*Salmonella* survives in phagosomes in primary human B cells in a growth arrested manner. *Salmonella* replicates in other cell types via a pathway involving activation of the kinase Akt1 [10]. In B cells, Akt-signaling is negatively regulated after BCR-triggering by Rap1 and Rap2 GTPases [31]. Whether this explains the control of *Salmonella* replication in human primary B cells remains to be established. An effective CD8^+^ cytotoxic T cell response is essential to control *Salmonella* infection [32], [33], possibly to eliminate *Salmonella* hiding inside cells. Our data show that also systemic spreading of *Salmonella* will be limited by elimination of the intracellular bacteria in B cells. Thus, vaccination strategies that aim to induce both *Salmonella*-specific antibodies and *Salmonella*-restricted CD8^+^ T cells may yield strong synergistic effects.

In view of coevolution of pathogens and the eukaryotic immune system, it is striking that *Salmonella* abuses the specificity of the adaptive immune system. Previously, it was shown that cells of the innate immune system and erythrocytes can be hiked by pathogens for systemic spreading of infection within immunocompetent hosts. Examples include HIV (DCs) [34] and *P. falciparum* (erythrocytes) [35]. The adaptive immune system has evolved to clear infections, while simultaneously generating immunological memory to ensure rapid immunity against reinfection. We now show that pathogen-recognizing and antigen-specific immune cells from the adaptive immune system can be applied by bacteria to escape direct recognition by other immune cells and to mediate bacterial dissemination. Although the adaptive immune system responds to pathogens to limit infection, some pathogens, as illustrated here for *Salmonella,* have adapted to this and use the specific aspect in immune responses for survival and systemic spreading.

## Supporting Information

Figure S1 **GFP-** ***Salmonella*** **distribution per cell after 0 and 18 hours.** B cells were infected with anti-BCR coated GFP-expressing *Salmonella* and the number of intracellular *Salmonella* was analyzed by ImagestreamX. Using IDEAS spot-count feature the numbers of intracellular *Salmonella* were discriminated by either one, two or three and more *Salmonella* per cell. Shown are examples of *Salmonella* count from one representative experiment of two independent experiments.
(TIF)

Video S1 **GFP-** ***Salmonella*** **growth in Ramos B cells.** Widefield fluorescence microscopy of living cells at 37°C confirms the results from the FACS experiment in Fig. 2 B. Left panel: GFP signal, right panel: GFP signal projected on the transmission image, taken every 30 min. Bacterial growth is observed in a Ramos B cell that has phagocytosed anti-human BCR antibody coated GFP-expressing *Salmonella*. Total duration: 11.5 hours.
(MOV)

Video S2 **GFP-** ***Salmonella*** **does not grow in B cells.** Widefield fluorescence microscopy of living primary human B cells at 37°C confirms the results from the FACS experiment in Fig. 2 B. Left panel: GFP signal, right panel: GFP signal projected on the transmission image, taken every 20 min. No growth of GFP-*Salmonella* is observed in primary B cells. Total duration: 14.5 hours.
(MOV)

Video S3 **GFP-** ***Salmonella*** **growth is actively suppressed in human primary B cells.** Widefield fluorescence microscopy of living cells at 37°C as in Fig. 2 C. Left panel: GFP signal, right panel: GFP signal projected on the transmission image. Images taken every 30 min. Cells were treated with Edelfosine, an apoptosis-inducing alkyl-lysophospholipid. No bacterial growth is observed in primary B cells until the cell undergoes apoptosis after 2 h, showing clear apoptosis associated morphological changes of the nucleus and membrane blebbing. After the onset of apoptosis, *Salmonella* expansion is observed within the apoptosing cell. Total duration: 15 hours.
(MOV)

Video S4 **GFP-** ***Salmonella*** **containing human primary B cells show invasive behavior in a monolayer of CD40L-expressing 3T3 fibroblasts.** A co-culture of living primary human B cells infected with GFP-expressing *Salmonella* and CD40L-expressing 3T3 cells was imaged using widefield fluorescence microscopy at 37°C. Depicted is the GFP signal projected on the transmission image with images taken every 30 min. The B cells repeatedly move under and over the monolayer. Arrows indicate the B cell, white: above the monolayer, black: under the monolayer. This Movie demonstrates the improved survival of B cells when co-cultured with CD40L expressing 3T3 cells. Also, excretion of the bacterium towards the end of the Movie (best visible at 12 h) is observed although the *Salmonella* is still contacting the B cell. Total duration: 15 hours.
(MOV)

Video S5 **GFP-** ***Salmonella*** **is excreted from primary human B cells.** Living primary B cells infected with GFP-expressing *Salmonella* and co-cultured with CD40L-expressing 3T3 cells were imaged using widefield fluorescence microscopy at 37°C. Depicted are GFP and Texas-Red signals projected on the transmission image with images taken every 30 min. A single primary B cell that has phagocytosed anti-BCR coated GFP-expressing *Salmonella* is followed over time in medium containing anti-LPS antibodies labeled with Texas-Red. The bacterium is protected from staining by the extracellular antibodies as long as it resides intracellular. Double labeling is observed after 6.5 hr, showing access of the B cell-associated *Salmonella* to the antibody-containing medium. Total duration: 11.5 hours.
(MOV)

Video S6 **GFP-** ***Salmonellae*** **excreted from human primary B cells are capable of infecting 3T3-CD40L fibroblasts.** Living primary human B cells infected with GFP-expressing *Salmonella* were co-cultured with CD40L-expressing 3T3 cells, and imaged by widefield fluorescence microscopy at 37°C. Depicted are GFP and Texas-Red signals projected on the transmission image. Images are collected every 30 min. 5 hours after the onset of the experiment a single human primary B cell infected with a GFP-expressing *Salmonella* moves from a distant location into the viewing plane from the upper right corner. The bacterium is excreted from the B cell and subsequently infects the 3T3-CD40L monolayer followed by rapid growth of *Salmonella* in the 3T3-CD40L cells. This further demonstrates that growth of the bacterium is actively suppressed inside the human primary B cell, while its viability is maintained. Primary B cells thus form a survival niche for *Salmonella*. Total duration: 15 hours.
(MOV)
