# Supplementary figures and images for: Selective Infection of Antigen-Specific B Lymphocytes by Salmonella Mediates Bacterial Survival and Systemic Spreading of Infection
Source: PLoS One. 2012 Nov 29;7(11):e50667. doi: 10.1371/journal.pone.0050667 (PMC3510171; doi:10.1371/journal.pone.0050667)

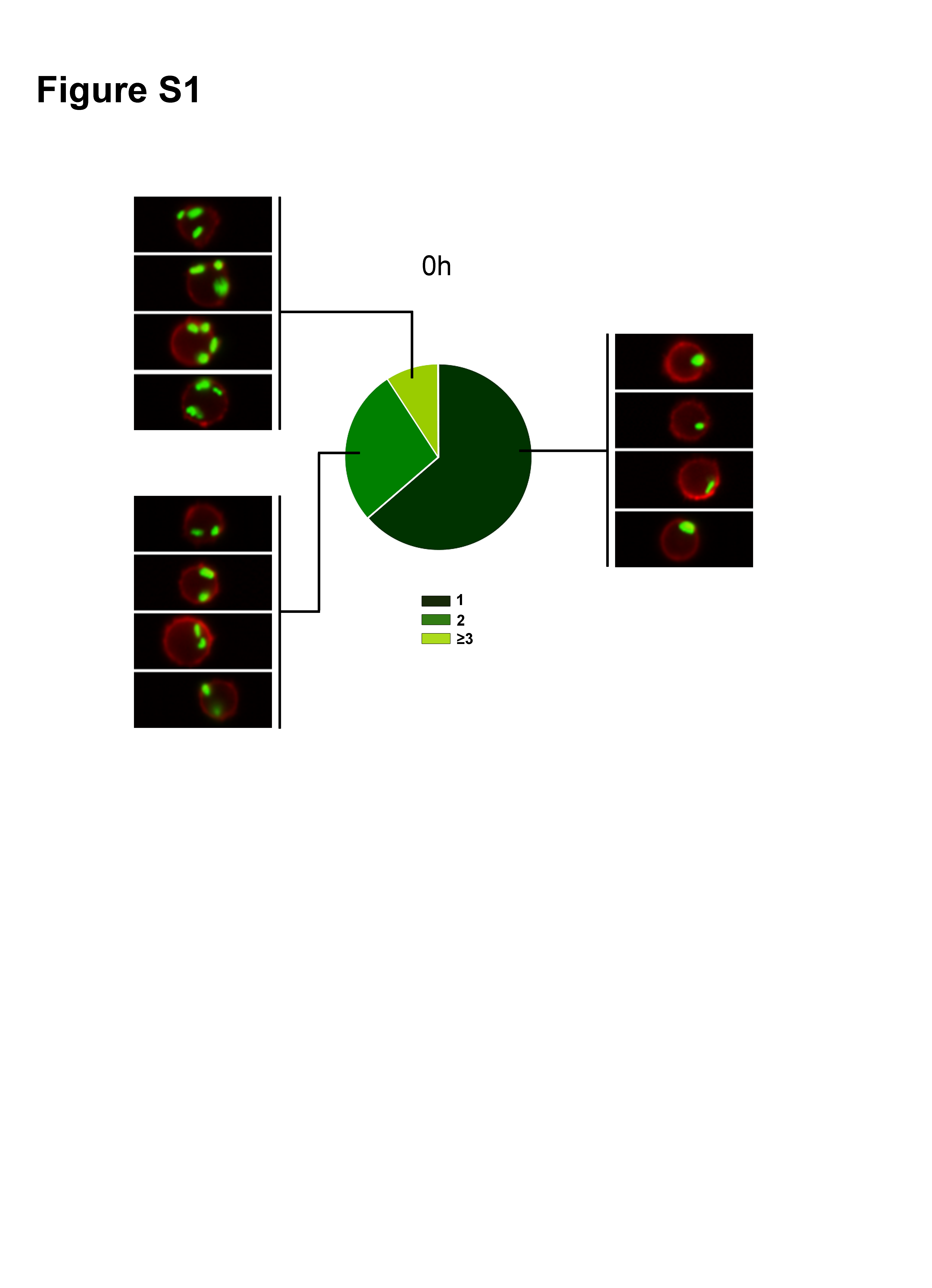

Supplement: Figure S1 — GFP- Salmonella distribution per cell after 0 and 18 hours. B cells were infected with anti-BCR coated GFP-expressing Salmonella and the number of intracellular Salmonella was analyzed by ImagestreamX. Using IDEAS spot-count feature the numbers of intracellular Salmonella were discriminated by either one, two or three and more Salmonella per cell. Shown are examples of Salmonella count from one representative experiment of two independent experiments. (TIF) [file pone.0050667.s001.tif]
